# Beyond effectiveness of the Strengthening Families Program (10-14): a scoping RE-AIM-based review

**DOI:** 10.1186/s41155-021-00182-z

**Published:** 2021-06-15

**Authors:** Nádia P. Pinheiro-Carozzo, Sheila G. Murta, Luís Gustavo do A. Vinha, Isabela M. da Silva, Anne Marie G. V. Fontaine

**Affiliations:** 1 Departamento de Psicologia, Centro de Ciências Humanas, Universidade Federal do Maranhão, Cidade Universitária Dom Delgado, Avenida dos Portugueses, 1966, Bacanga, São Luis, MA 65080-805 Brazil; 2 Departamento de Psicologia Clínica, Instituto de Psicologia, Universidade de Brasília, Campus Universitário Darcy Ribeiro, Brasília, DF 70910-900 Brazil; 3 Departamento de Estatística, Instituto de Ciências Exatas, Universidade de Brasília, Campus Universitário Darcy Ribeiro, Brasília, DF 70910-900 Brazil; 4 Faculdade de Psicologia e de Ciências da Educação, Universidade do Porto, Rua Alfredo Allen, 4200-135 Porto, Portugal

**Keywords:** Drug abuse prevention, Family intervention, Program evaluation, Strengthening Families Program SFP (10-14), RE-AIM

## Abstract

A scoping review, based on the RE-AIM framework, was conducted to analyze evidence of reach, effectiveness, adoption, implementation, and maintenance of the Strengthening Families Program (10-14), a preventive family-based substance abuse program for adolescents. Sixty-five articles were included. The results disclosed that effectiveness, implementation, and maintenance at the individual-level were the most evaluated aspects, while reach, maintenance at the setting-level, and adoption were the least investigated aspects. Positive effects on drug abuse prevention and protective parenting factors were found in the U.S. studies. Likewise, Latin American studies have shown the improvement of parenting practices. However, European studies have produced mixed results, with predominantly null effects on substance abuse. The implementation quality was high. There is no available evidence of adoption and maintenance at the setting-level by the organizations that implemented it. New studies must examine the reach, adoption, and sustainability of the program to lay foundations for its future use as an instrument of public policies.

**Supplementary Information:**

The online version contains supplementary material available at 10.1186/s41155-021-00182-z.

## Introduction

Prevention is considered the cornerstone of substance abuse approaches (United Nations Office on Drugs and Crime, 2014). The literature indicates that preventive interventions that include the family—as opposed to individual or parental-only interventions—are more effective at preventing drug abuse (Foxcroft, Ireland, Lister-Sharp, Lowe, & Breen, 2003; Kumpfer, Magalhães, & Xie, 2017). One example of family-based intervention is the Strengthening Families Program—SFP (10-14), an intervention developed in the United States—U.S. (Kumpfer, Molgaard, & Spoth, 1996).

SFP (10-14) is a universal prevention program, therefore aimed at the entire population, regardless of the degree of exposure to risky factors (Weisz, Sandler, Durlak, & Anton, 2005). The program targets teenagers aged 10 to 14 and their parents/caregivers (Kumpfer et al., 1996) and it is based on the Theory of Social Learning, Theory of Social Ecology, and Theory of Family Systems. It comprises seven weekly 2-h meetings sessions. During the first hour, parents and adolescents attended different sessions, and in the second hour, they together attended a family-session (Kumpfer, 2014). Among the primary outcomes, SFP aims to reduce child mistreatment, substance abuse, delinquency, and school failure. The secondary outcomes include improving parenting practices and parent-child relationship quality, as well as developing effective parenting (Kumpfer et al., 2015).

In addition to the U.S., this program was implemented in several European and Latin American countries. Systematic reviews that analyzed the effectiveness evaluation of this intervention using American samples, indicated that SFP (10-14) was one of the efficacious family-based interventions for preventing marijuana use (Gates, McCambridge, Smith, & Foxcroft, 2006), and promising for preventing alcohol use (Foxcroft et al., 2003; Foxcroft & Tsertsvadze, 2011). According to the systematic review performed by Ladis et al., 2019, SFP (10-14) met most of the efficacy criteria of the Society for Prevention Research (Gottfredson et al., 2015).

However, in contrast to the initial positive findings, recent studies performed by independent groups, in the U.S. and Europe, showed SFP (10-14) lacked effectiveness in one of the primary outcomes: substance abuse. The discrepancy in effectiveness between the initial and recent studies, called the decline effect, may be related to the program’s content, which may be effective for some families living in specific contexts but not for others; the randomness of the findings; the adoption of data analysis procedures different from those in the original studies; or failure to replicate the main components of SFP (10-14). Therefore, the generalization (external validity) of SFP (10-14) results in the world remains an important research gap (Gorman, 2017).

The inconsistency between the findings of the initial studies and the recent SFP (10-14) studies indicates that it is relevant to investigate how, for whom, at what costs, and for how long this intervention has been implemented in the world. Understanding these elements can help elucidate the impact on public health and the SFP (10-14) translation process in practice (Type 2 Translation). Type 2 Translation “involves the translation of program development to implementation (i.e., efficacy trials with emphasis on internal validity and effectiveness trials with emphasis on internal and external validity)” (Fishbein, Ridenour, Stahl, & Sussman, 2016, p. 7) and helps establish evidence-based interventions (Fishbein et al., 2016). However, it is one of the most deprived targets in the field of prevention science (Spoth, Rohrbach, et al., 2013; Spoth, Trudeau, et al., 2013) and family and parental programs, in particular (Mauricio, Gonzales, & Sandler, 2018).

The RE-AIM framework is a framework that allows understanding the process through which evidence-based interventions are adopted, implemented, and sustained on a large scale (Glasgow, Vogt, & Boles, 1999). RE-AIM is an acronym for the dimensions: Reach, Effectiveness, Adoption, Implementation, and Maintenance, both at the individual-level and at the setting-level (More information can be found at www.re-aim.org).

This framework has been widely used in studies seeking to understand real-world implementations, impacts, and the chance of generalization or replicability of the program to other groups and settings (Boersma, van Weert, Lakerveld, & Dröes, 2015; Cuthbert, King-Shier, Ruether, Tapp, & Culos-Reed, 2017; McGoey, Root, Bruner, & Law, 2015; Schlechter, Rosenkranz, Guagliano, & Dzewaltowski, 2016). Although specific RE-AIM dimensions may be used (Glasgow et al., 2019), comprehensive studies that include the five dimensions provide a holistic view of the topic of interest and help address “which complex intervention for what type of complex patients, delivered by what type of staff will be most cost-effective, under which conditions and for what outcomes” (Gaglio, Shoup, & Glasgow, 2013, p. e45).

### The present study

This study addresses an important knowledge gap regarding the translation of SFP (10-14) into practice and public health impact. It aims to capture all five dimensions of the RE-AIM framework related to SFP (10-14) by addressing the question: What is the evidence about the reach, effectiveness, adoption, implementation, and sustainability of the Strengthening Families Program (10-14) around the world? It therefore seeks to expand, in two different ways, the scope and findings of Gorman’s (2017) review. The first way is by adding findings of other outcomes, beyond substance abuse. The second way is to add to the effects other dimensions that show SFP’s impact on public health, i.e., reach, adoption, implementation, and maintenance at the setting- and individual-level.

The main purpose of this study was to analyze the evidence of the reach, effectiveness, adoption, implementation, and maintenance of the SFP (10-14). The specific objectives were to verify the extent to which RE-AIM components have been reported in the SFP assessment literature; analyze the populations the program reached and through which strategies; the course used by the services and implementation agents for the adoption and implementation of the program; evidence for iatrogenic effects and effectiveness identified in studies referring to substance abuse, behavioral problems, and familial and academic outcomes; and finally, evidence regarding the maintenance of effects among individuals and of the implementation of SFP (10-14) among organizations.

## Methods

### Study design

This study is a scoping literature, which in an exploratory way, maps the literature on a topic identifying key concepts, research findings and gaps (Arksey & O'Malley, 2005). Together with RE-AIM framework approach, the inclusion of a range of methodological designs was possible to understand “how” and “why” the REAIM outcomes are generated (Glasgow et al., 2019; Holtrop, Rabin, & Glasgow, 2018). Furthermore, although we are not proposing a systematic review or meta-analysis, we followed most statements of the PRISMA protocol (Shamseer et al., 2015).

### Eligibility criteria

A systematic search was conducted to identify aspects of reach, effectiveness, adoption, implementation, and maintenance of the 7-session SFP 10-14, without country or date restrictions. Inclusion criteria were (I) articles published in peer-reviewed indexed journals, with experimental, quasi-experimental or non-experimental design and with quantitative, qualitative, or mixed analyses; (II) published in English, Portuguese, or Spanish; and (III) articles about the assessment of SFP’s implementation process, effectiveness, efficiency, and/or sustainability. Exclusion criteria were (I) review, theoretical, or case-study articles; (II) articles exclusively about SFP’s adaptation process before its implementation, as they did not offer data about SFP evaluations, that is, articles describing SFP’s surface-structure and/or deep-structure adaptations, analyzing the quality or adequacy of adapted materials or content to be included (thus, studies that focused on the adaptation process after SFP implantation and which added information about any RE-AIM dimensions, were included); and (III) articles that presented all results of SFP combined with another intervention in a way that prevented any RE-AIM analysis independent of the program.

### Information sources

The initial search of the article was performed on Lilacs, Medline, PsycINFO, PsycArticles, PubMed, Scopus (Elsevier), and Web of Science databases. December of 2019 was the end date. Subsequently, reference sections of the articles previously retrieved, and publications listed on the program’s official website were examined to identify additional articles.

### Search strategy

The search terms were: [(“*strengthening families program*” OR “*strengthening families programme*” OR SFP) AND (“*process evaluation*” OR *reach* OR *effectiveness* OR *efficacy* OR *adoption* OR *implementation* OR *maintenance*)] which could be present in any part of the article.

### Selection process

**Fig. 1:**
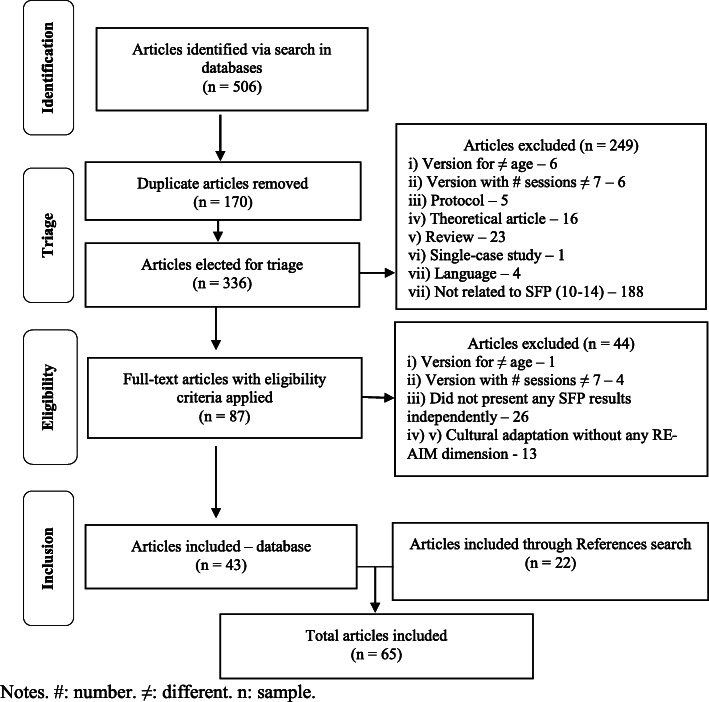
Selection steps to compose the basis of the document. #: number. ≠: different. n: sample

The selection procedure is illustrated in Fig. 1.

### Data items

A conceptualization form, including article characteristics, information about SFP (10-14), and components of RE-AIM framework—based on Kessler et al. (2013)—was used for data extraction.

### Data synthesis

Numerical data, such as the number of follow-ups assessments, and attrition rates, were treated by descriptive statistics. Mean and standard deviation was calculated. Non-numerical data—data that deal with descriptions rather than numbers—were analyzed by thematic analysis (Braun & Clarke, 2006). Accordingly, we identified themes that were allocated to and developed in an analytic framework, elaborated in the previous step.

## Results

### Documental basis

The documental basis consisted of 65 articles (highlighted with an asterisk in the References section). Articles were published between 1996 and 2019. Fifty studies were conducted in the U.S., eleven in Europe, and four in Latin American. Most of the studies adopted an experimental design (35 articles, 54%). An analysis of the objectives revealed that effectiveness, as well as maintenance at the individual-level, and implementation, in this order, are the most evaluated aspects. Investigating the adoption of SFP (10-14) and its maintenance at the setting-level were not a primary goal for any of the studies. Beyond these main objectives, some articles indicated or analyzed other RE-AIM components within their scope, treating them as secondary objectives or descriptions of SFP (10-14).

### The version of SFP (10-14) analyzed

A large number of articles used a logic model to describe the mechanism of change expected from SFP (10-14). In 29 of the articles (45%), the logic model components were presented in textual format, 4 (6%) in a diagram or table, and 5 (8%) in both text and diagram or table.

Of the studies, 74% recruited SFP (10-14) participants in schools; 6% in health services; 3% in social assistance services; 2% in both health and social assistance services; 5% in other services (such as community centers and religious institutions); and 9% of the studies did not mention the recruiting location(s). As for the implementation setting, 49% of the articles did not report it, 42% implemented SFP in schools, 3% in social assistance institutions, 3% in health institutions, and 2% in other institutions, such as community centers. The article dealing with secondary data included studies with various recruitment and implementation sites (Hill, Cooper, & Parker, 2019).

Only 21 articles (32%) provided information about the program’s implementation agents. This included (a) education: bachelor’s degree (Cantu, Hill, & Becker, 2010; *Coatsworth et al., 2015; Coatsworth, Timpe, Nix, Duncan, & Greenberg, 2018; *Coatsworth, Duncan, Greenberg, & Nix, 2010; *Lindsay & Strand, 2013; *Riesch et al., 2012), health education professionals (*Aalborg et al., 2012; *Byrnes, Miller, Aalborg, & Keagy, 2012; Byrnes, Miller, Aalborg, Plasencia, & Keagy, 2010), nurses (*Semeniuk et al., 2010; Vasquez et al., 2010), and teachers (*Corea et al., 2012; *Skärstrand, Sundell, & Andréasson, 2014); (b) skills: experience working with youth, parents, or families (*Coatsworth et al., 2010, 2015, 2018), being bilingual (*Orpinas et al., 2014; *Reidy, Orpinas, & Davis, 2012), experience working with the Latino population (*Orpinas, Reidy, et al., 2014; *Reidy et al., 2012), community, health care, or social agency workers (*Coombes, Allen, Marsh, & Foxcroft, 2009; *Guyll, Spoth, Chao, Wickrama, & Russell, 2004; *Segrott et al., 2017; *Spoth, Guyll, Lillehoj, Redmond, & Greenberg, 2007); (c) specific SFP training (*Aalborg et al., 2012; *Byrnes et al., 2012; *Coatsworth et al., 2010, 2015, 2018; *Corea et al., 2012; *Hill et al., 2019; *Orpinas et al., 2014; *Reidy et al., 2012; *Riesch et al., 2012; *Segrott et al., 2017; *Semeniuk et al., 2010; *Skärstrand et al., 2014); (d) experience with other SFP implementations and (e) gender: 82% female (*Segrott et al., 2017).

Three types of session structures were identified: (1) a little over half the articles (53%) reported using the seven weekly sessions version, which is 2-h long—separate 1-h meetings for parents and adolescents, and 1 h for the family (joint meeting with both parents and adolescents); (2) around one-third (33%) used the seven weekly meetings version—consisting of six sessions of 1-h meetings for parents and adolescents and 1 h for the family, while the seventh and last session was a joint/family meeting of 1 h; (3) the smallest proportion (3%) used a composite version, comprising two parts: the first part had seven weekly meetings, with six sessions of separate one to one-and-a-half-hour meetings for parents and adolescents, and the seventh and last session was a 1-h family meeting; in addition, the second part consisted of five sessions, originally designed to be booster meetings, but inserted as standard sessions, four of which were separate 1-h meetings for parents and adolescents, while the fifth was a 2-h family session (*Skärstrand, Bränström, Sundell, Källmén, & Andréasson, 2009; *Skärstrand et al., 2014). Seven articles (11%) gave incomplete or no information about their structure of choice.

Follow-up sessions were carried out in 64% of the studies. Among these, the number of follow-up sessions varied from 0 to 12, with an average of 2.9. The first follow-up session took place between 3 and 18 months after SFP (10-14), and the last between 24 and 84 months after SFP (10-14). Three studies conducted four booster sessions (*Baldus et al., 2016; *Bröning et al., 2017; *Segrott, 2013); however, only one reported when the sessions took place: between 4 and 6 months after SFP (10-14) (*Bröning et al., 2017). Two studies transformed the booster sessions into standard sessions (*Skärstrand et al., 2009; *Skärstrand et al., 2014).

### Reach of the SFP (10-14)

Although six studies were to understand some aspect of the reach, only one study reported on all Reach criteria (*Byrnes et al., 2012) (cf. Supplementary Appendix). The exclusion criteria for participants of the various studies focused on the characteristics of families and community. Adolescents in alcohol, tobacco, and other drugs (ATOD) treatment were excluded (*Aalborg et al., 2012; *Byrnes et al., 2010, Byrnes et al., 2012), as well as those who did not live with their parents (*Byrnes et al., 2012). Families not fluent in English (*Aalborg et al., 2012; *Byrnes et al., 2012), with parents who did not self-identify as Latinos (*Orpinas, Reidy, et al., 2014; *Reidy et al., 2012) or who did not speak Spanish, were excluded from the studies with Spanish-speakers living outside their home country (*Orpinas, Reidy, et al., 2014; *Reidy et al., 2012). Families from communities with an employment rate of more than 50%, where there was the availability of a preventive ATOD program, or with a member who had a university degree (*Spoth, Clair, Greenberg, Redmond, & Shin, 2007; *Spoth, Guyll, et al., 2007), were also excluded.

The number of participants in the experimental group (EG) was given as numbers of parents and adolescents or number of families. In the seven studies (11%) that provided the number of parents and adolescents, it ranged from a minimum of 13 parents and 15 adolescents to a maximum of 151 parents and 144 adolescents (in the pre-test). In the 39 studies (60%) that listed the number of families, the minimum was 12 and the maximum was 238, in the pre-test. Other articles did not provide the number of participants in the pre-test, or provided it for just one group, that is, either parents or adolescents.

Additionally, a percentage or the representativeness of the target population reached was not determined, since there was discrepancy in the adopted denominator, which was alternately (a) the number of invited adolescents or families (Errasti Pérez et al., 2009; *Segrott et al., 2017; *Semeniuk et al., 2010; *Trudeau, Spoth, Randall, Mason, & Shin, 2012; *Riesch et al., 2012) or (b) the number of eligible adolescents or families (Bamberger, Coatsworth, Fosco, & Ram, 2014; *Bröning et al., 2017; *Byrnes et al., 2012; *Chilenski, Welsh, Perkins, Feinberg, & Greenberg, 2016; *Lindsay & Strand, 2013; *Mason, Chmelka, Trudeau, & Spoth, 2017; *Skärstrand et al., 2014; *Spoth, Shin, Guyll, Redmond, & Azevedo, 2006; *Spoth, Clair, & Trudeau, 2014, *Spoth, Trudeau, Shin, Randall, & Mason, 2019; *Whitesell et al., 2019).

Regarding the participants’ characteristics, in 42 (65%) of the articles, the participants were low-income and eligible for social programs. In 40 (61%) articles, the households were two-parent families, and in 41 (63%) articles, families had an average of three children, with parents who had completed a high-school education. Despite being a minority, it is notable that, in three of the studies, the adolescents had a statement of special needs education or had experienced behavioral problems (*Lindsay & Strand, 2013), had hyperactivity (100% of the adolescents) and conduct problems (90%) (*Coombes et al., 2009), or were living in a community with high levels of social problems (Vasquez et al., 2010).

The methods used to recruit participants were personal invitation (10 articles, 15%), invitation addressed and mailed to residence (10 articles, 15%), phone call (5 articles, 8%), leaflets and/or flyers (3 articles, 5%), and others (2 articles, 3%). One article reported difficulty encountered during the recruitment: the recruiting agency was not the agency that implemented the program, because the latter would only invite “at-risk” families, while SFP (10-14) is a proposal for universal intervention (*Segrott et al., 2017). Two articles identified procedures that facilitated recruitment: a community leader carrying it out (*Orpinas, Reidy, et al., 2014), working to build bonds with the families since the invitation moment, and emphasizing that the program is not about judging the family’s resources or deficit, but rather about strengthening and solutions (*Segrott, 2013).

One article listed strategies for reducing obstacles to participation: reimbursing transportation expenses, offering games to non-participants accompanying the participants, and non-alcoholic beverages (*Segrott, 2013). Two other articles identified elements that influenced the family’s participation in SFP (10-14): the availability of childcare, a convenient place and time, affinity with the community, transportation, refreshment, payment, and the number of instruments to fill out (*Reidy et al., 2012). Thus, the following suggestions were emphasized: consider the benefit-cost ratio for families, simplify communication by explaining the study, training more implementation agents, and deliver the program in multiple services (education, health, social) to broaden its reach (*Segrott et al., 2017).

One article that investigated the recruiting process reached several conclusions. Specifically, (a) African-Americans were more likely to accept the invitation and show up at the meetings than Asians; (b) families from communities with high unemployment levels were less likely to participate; (c) families headed by single women presented a higher chance of participation; (d) Caucasians, Asians, people with a college degree, and older parents tended to participate more actively in the activities than African-Americans; and (e) the level of participation dropped by 44% with increasing high school dropout rates (*Byrnes et al., 2012).

### Effectiveness of the SFP (10-14)

Due to the peculiar characteristics of effectiveness, we decided to present the data on a regional basis, the regions being the U.S., which contains most of the studies; Europe, represented by Germany, Spain, Poland, UK, and Sweden; and Latin America, represented by Bolivia, Chile, Colombia, Ecuador, Honduras, and Panama. Additionally, the data were organized so that the primary outcomes are presented first and then the secondary outcomes. Furthermore, to facilitate the reader’s understanding of the program’s effectiveness, data regarding maintenance at the individual-level—outcomes maintained for 12 months or more—were reported in this section. None of the mentioned studies reported data about broader outcomes, iatrogenic, or side-effects.

#### The U.S.

Plenty of evidence indicated the effectiveness of SFP (10-14) in the U.S., for several outcomes. Among the primary outcomes: delaying the use of alcohol and other drugs (e.g., *Spoth, Redmond, & Lepper, 1999; *Spoth, Reyes et al., 1999; *Spoth, Redmond, Shin, & Azevedo, 2004, *Spoth, Clair, Shin, & Redmond, 2006; Spoth, Shin, et al., 2006; *Spoth, Trudeau, Guyll, Shin, & Redmond, 2009), decreasing exposure to substance use (*Spoth et al., 2012), prevention of new users (*Spoth, Redmond, & Shin, 2001), a long-term decrease of drug abuse (e.g., *Spoth, Trudeau, et al., 2009; *Spoth et al., 2014), misuse of medications (*Spoth, Trudeau, Shin, & Redmond, 2008), long-term academic success, and school engagement (*Spoth, Randall, & Shin, 2008), and also effects on non-participating adolescents (Rulison et al., 2015). Moreover, some of the secondary outcomes were positive affect, support, involvement, and closeness (*Coatsworth et al., 2015), as well as direct and indirect effects on the improvement of parenting practices (e.g., *Cantu et al., 2010, *Coatsworth et al., 2010, *Orpinas, Reidy, et al., 2014, *Redmond, Spoth, Shin, & Lepper, 1999, *Spoth, Redmond, & Shin, 1998), affection (*Spoth et al., 1998), cohesion and family involvement (e.g., *Chilenski et al., 2016, *Riesch et al., 2012), and fewer depression-related symptoms in adulthood (*Mason et al., 2017, *Trudeau, Spoth, Randall, & Azevedo, 2007). Long-term decrease of antisocial behaviors (*Spoth, Redmond, & Shin, 2000), and risky sexual behavior (*Spoth et al., 2014), long-term positive relationship (*Spoth et al., 2019), and better problem-solving skills in the medium term (*Semeniuk et al., 2010) were also observed.

#### Europe

Mixed results were found in Germany and the UK; positive results in Spain; and null results in Poland and Sweden. In Germany, no significant difference was found concerning the following primary outcomes: first use of drugs, drug use in the past 30 days, the lifelong use of alcohol and marijuana (*Baldus et al., 2016), and general drug use (*Bröning et al., 2017). On the other hand, considering secondary outcomes, significant improvement in children’s behavioral problems (*Bröning et al., 2017), but no significant improvement concerning behavioral problems was found (*Baldus et al., 2016).

In the UK, when dealing with quantitative measures, the results indicated (a) significant decrease in secondary outcomes, such as child conduct problems (*Lindsay & Strand, 2013), youth difficulties in communication and emotional management, and parent emotional symptoms ((*Coombes et al., 2009); (b) significant decrease in the primary outcomes, such as misuse of alcohol and drugs (*Coombes et al., 2009); and (c) increase in secondary outcomes: parents’ mental well-being, parenting skills (*Lindsay & Strand, 2013), parenting limit setting, and prosocial behavior (*Coombes et al., 2009). However, one study reports no significant changes, both on primary and secondary outcomes. Nonetheless, when dealing with qualitative measures, improvements in secondary outcomes were reported, such as (a) in adolescent emotions, positive peer interactions, and communication and family interaction; and (b) family functioning (*Coombes et al., 2009).

In Spain, significant differences were found concerning both primary outcomes, in the use of drugs in the past 30 days, and secondary outcomes, as improvements were detected for the “parental attitudes in response to youth alcohol use” and “bonds between parents and children” when families participated in 8 sessions (seven sessions plus one booster) (Errasti Pérez et al., 2009). Both in Poland (*Foxcroft, Callen, Davies, & Okulicz-Kozaryn, 2017) and in Sweden (*Skärstrand et al., 2014), no significant differences were found, either concerning primary outcomes, for use of alcohol, tobacco, or other drugs; or secondary outcomes, which include parent-child relationships, parenting practices, and child behavioral problems in the Polish assessment and defiant behaviors in the Swedish assessment.

#### Latin America

Regarding secondary outcomes, decreases in yelling, insulting, and loss of control in light of adolescent bad behavior were reported in Chile (*Corea et al., 2012); improvements in parenting practices and parental self-esteem in Honduras (Vasquez et al., 2010); positive changes in adolescent behavior, parenting practices, marital and family relationship in Panama (*Mejia, Ulph, & Calam, 2015); improvement in positive parenting and parental hostility in Colombia and Bolivia; and in parental involvement, consistent discipline, parental monitoring, and parental communication about risky behaviors in Ecuador (*Orpinas, Ambrose, et al., 2014). Concerning primary outcomes, either null results were found in Chile (*Corea et al., 2012) and Honduras (Vasquez et al., 2010), or they were not evaluated such as in Bolivia, Colombia, and Ecuador (*Orpinas, Ambrose, et al., 2014) and in Panama (*Mejia et al., 2015).

### Adoption of the SFP (10-14)

None of the articles aimed to understand the SFP (10-14) adoption. Furthermore, in 97% of the analyzed articles, no information was provided about the criteria in this dimension. Only two studies gave information about the criteria of service adoption (*Aalborg et al., 2012; *Segrott et al., 2017) and three about adoption by implementation agents (*Aalborg et al., 2012; *Orpinas, Reidy, et al., 2014; *Segrott et al., 2017). The data supplied information about the characteristics of the services or implementation agents who participated in the study, and about barriers and implementation agents for their adoption of SFP (10-14). No data were supplied about services or implementation agents who had been excluded from the study or not invited to participate in it. This omission did not allow to calculate the adoption rate of the intervention.

Moreover, the characteristics and availability of adequate space of the services were considered to carry out the program (*Aalborg et al., 2012), as well as the implementation agents, their interest in offering SFP (10-14) (*Aalborg et al., 2012), bilingualism (English and Spanish), and experience in working with Latinos (*Orpinas, Reidy, et al., 2014). *Segrott et al. (2017) used Extended Normalisation Process Theory to understand the interaction of SFP (10-14) with its delivery systems. The results listed the funding availability to sponsor SFP (10-14) in the services, its incorporation into its routines, and recruitment and maintenance of the implementation agents as difficulties for adopting the program.

### Implementation of the SFP (10-14)

Fidelity was the most investigated implementation criterion. There were two criteria not reported in any article: uncounted intervention time and context.

#### Adaptations

In both studies by *Skärstrand et al. (2009, 2014), the focus age changed from 10 to 14, to between 12 and 14 years of age, content or items from the parent sessions were omitted, the parents’ presence was optional, the booster sessions were converted into standard sessions (renamed “part 2”), one extra session was added, family sessions were carried out only in the seventh session of part 1 and the fifth session of part 2, and the material prepared by the authors themselves about drugs and alcohol was added. Some of these modifications occurred due to practical problems, which prevented to simultaneously carry out the parent and adolescent sessions.

*Coatsworth et al. (2010) explained the creation of the Mindfulness-enhanced Strengthening Families Program (MSFP) that his research group evaluated in many articles. They argued that as original SFP already contained implicit principles of mindfulness, their “task was to make these implicit messages more explicit by adding short mindfulness activities and by altering some of the language within the program so that it more clearly reinforced principles and practices of mindfulness” (p. 04).

Other studies did not make any adaptations, but rather analyzed them. After analyzing implementation data from 97 SFP offers in the U.S., the authors concluded that adaptations in games, activities, random content, and group process accounted for 76% of all adaptation types; and that 79% of all adaptations made were justified by insufficient time, group attributes, and the number of participants (*Cooper, Shrestha, Hyman, & Hill, 2016). The inclusion of tribal language, as SFP was delivered to Indian-American families, social media, and substance use content did not impact SFP effectiveness in the short term (*Whitesell et al., 2019).

*Orpinas, Reidy, et al. (2014) did not introduce adaptations, but instead suggested some, based on an implementation experience with Mexicans living in the U.S.: (a) restricting group size; (b) more implementation agents per group; (c) fewer activities involving reading and writing; (d) presence of a translator; (e) offering information about drug use and sexual behavior; (f) addressing mistaken perceptions of cultural norms and expectations by age; (g) promoting appreciation of Latin culture; and (h) offering educational support for the community.

#### Dose

Two studies specifically included the dose criterion (*Riesch et al., 2012; *Segrott et al., 2017). *Riesch et al. (2012) defined the fully delivered dose as attending at least five of the seven sessions, while a partially delivered dose consisted of attending fewer than five. It was verified that a large number of family groups participating in the partial dose level received public assistance and reported being low-income. Meanwhile, in the full dose condition, parents reported being in a stable relationship. *Segrott et al. (2017) assessed dose delivery by the number of programs (and constituent sessions) delivered and dose received by the engagement of young people and parents/guardians. Another seven articles recorded the received dose using an attendance list (*Hill & Owens, 2013; *Orpinas, Reidy, et al., 2014; *Skärstrand et al., 2009; *Spoth, Guyll, & Day, 2002; Spoth, Guyll, Trudeau, & Goldberg-Lillehoj, 2002 *Spoth, Guyll, & Shin, 2009; *Spoth et al., 2014; *Trudeau et al., 2012).

#### Economic cost

Three articles carried out budget evaluations. They were all from the U.S. and focused on the drug abuse primary outcome. In the first publication, including the prevention of alcohol use, the intervention’s full cost was US$ 80,562, with 100 families per wave; the cost-effectiveness was US$ 12,459; the benefit-cost ratio was US$ 9.60 per US$ 1 spent; and the net-benefit was US$ 5923 per family (*Spoth, Guyll, & Day, 2002). In the second publication, for the prevention of methamphetamine use alone, the intervention cost was US$ 115,813 for the 117 families that received SFP; the cost per adolescent was US$ 990.00; the cost-effectiveness was US$ 25,385 to prevent each case; the benefit-cost ratio was US$ 3.84 per US$ 1 spent; the net benefit was US$ 2813 by the adolescent (*Guyll, Spoth, & Crowley, 2011). Moreover, in the third publication, although the objective was an economic analysis of PROSPER (Promoting School-University Partnerships to Enhance Resilience—a project that includes SFP and one school-based intervention), the authors reported that SFP’s cost varied between US$ 502 and US$ 572; the cost per participating family varied between US$ 278 and US$ 378; and the net benefit varied between US$ 6307 and US$ 6377 per family (*Crowley, Jones, Greenberg, Feinberg, & Spoth, 2012). Furthermore, one study detailed some intervention expenses, namely announcements to implementation agents, US$ 550.00; facilitator training, US$ 25,758; materials for the families, US$ 2,776; incentives for the family’s participation, US$ 13,620; website, US$ 5,385; intervention implementation, US$ 31,972; childcare, US$ 4620; and family transportation, US$ 445 (*Spoth, Guyll, & Day, 2002; Spoth, Guyll, Trudeau, & Goldberg-Lillehoj, 2002).

#### Fidelity

The documental basis contains different articles that published results from the same study/research project. Thus, the results reported here are those of the studies, instead of each article.

Most of the studies reported high fidelity indices. One study that compared SFP with family matters (FM) analyzed fidelity from two points of view: (a) adherence, that is, how much of the program was released as predicted in the handbook, and (b) “quality,” in other words, the implementation agents’ ability to conduct the intervention, for example, their enthusiasm and aptitude. The results indicated that, in SFP’s first offering, adherence was 66% and, from the third to the sixth offerings, it was 80%; while “quality,” in turn, remained stable at 66–67% across the offerings (*Aalborg et al., 2012). The average session adherence was 78% to 93%, having reached or exceeded the expected level (*Byrnes et al., 2010). A second study, which compared SFP to MSFP, analyzed fidelity from two different points of view: (a) adherence, which reached 89% and 93%, respectively; and (b) leader/facilitator effectiveness, concerning friendliness, acceptance, and clarity, which reached a mean M = 3.6 out of a maximum score of 4 or was classified as excellent, respectively (*Coatsworth et al., 2010, 2018).

A third study compared SFP to Preparing for the Drugs Free Years (PDFY) and analyzed fidelity under the adherence criterion, obtaining indices of 87% for family sessions, 83% for parent sessions, and 89% for adolescent sessions (e.g.: *Spoth, Redmond, & Lepper, 1999, Spoth, Clair, et al., 2006, Spoth, Shin, et al., 2006, Spoth, Randall, & Shin, 2008, Spoth, Trudeau, et al., 2008, Spoth, Trudeau, et al., 2009; *Trudeau et al., 2007), with an 85% average (e.g.: *Spoth, Trudeau, et al., 2008). In the PROSPER study, SFP’s fidelity surpassed 90% (*Spoth, Clair, et al., 2007). Finally, one study analyzed the UK’s SFP version, indicating adherence from 90 to 99% (*Segrott et al., 2017). Furthermore, when delivered to Indian-American families, fidelity was over 90%, across youth, adult, and family sessions (*Whitesell et al., 2019).

Three studies (5%) analyzed fidelity predictors, and also reported its level. The results indicated that fidelity was strongly and inversely related to the number of families in each SFP (10-14) offering and the years of experience of the implementation agents; and was strongly and positively related to the number of implementation agents, with the same minority status of the participant and facilitator (*Cantu et al., 2010). Fidelity was better evaluated when families were chosen to participate in SFP (10-14) than when they chose to participate. It was positively related to adolescent satisfaction but negatively related to parent satisfaction. Thus, the authors recommended medium level fidelity as ideal, since high fidelity implies low flexibility, and low fidelity implies a failure to deliver the core components (*Byrnes et al., 2010). When correlating fidelity from the point of view of the program’s content components (i.e., didactical instructions about the expected behaviors, communication, increase in family identity, and cohesion) and process components (i.e., facilitator’s actions when delivering the components, for example, encouraging discussion, availability of materials, etc.), with the expected parenting practice results, it was discovered that the majority of correlations were not significant for European-Americans, but were significant for participating ethnic minorities (*Hill & Owens, 2013).

One study verified whether the implementation adherence and quality of implementation (composed by (1) group engagement, (2) group participation, and (3) quality of delivery, that is “both positive and negative features of facilitators’ behavior” p. 417) had been maintained for 6 years. The results indicated that adherence, as well as group engagement, group participation, and the quality of delivery, were highly maintained over the period (*Spoth, Guyll, Redmond, Greenberg, & Feinberg, 2011).

#### Engagement

Four articles (6%) reported engagement or active participation in the group. The results demonstrated (a) a score of 3.7 out of a maximum of 4 and an excellent classification, both were monitored through an analysis carried out by trained observers (*Coatsworth et al., 2010, 2018, respectively); (b) high scores in 94% of the 22 delivered offerings analyzed by the implementation agents (*Segrott et al., 2017); (c) a high level of engagement in the first session (i.e., involvement, interest, resistance, and positive affect toward the implementation agents and group members) positively correlated with the parent’s educational level and parental involvement; and (d) an increase in engagement level throughout of the sessions had a positive correlation with session attendance and with a companion, and a negative correlation with baseline measurements of negative affect and parental avoidance (Coatsworth, Hemady, & George, 2017).

Additionally, two articles analyzed engagement. Bamberger et al. (2014) verified that engagement increased over time, linearly with some deceleration, and aspects of family tension were related to both initial levels and session-to-session changes in engagement. *Elreda, Coatsworth, Gest, Ram, and Bamberger (2016) analyzed the relationship between group process and participant progress and intervention outcomes. They concluded that participants, who were better connected and reciprocated, experienced greater participant progress. Moreover, regarding youth, discomfort in group experience was negatively correlated with self-worth and mastery, and connectedness was negatively correlated with internalizing symptoms. Regarding mothers, discomfort across sessions was negatively correlated with negative affective quality of the mother-adolescent relationship, recurring mother-adolescent conflict, use of harsh discipline, and self-judgment. On the other hand, connectedness was positively correlated with emotional awareness during parenting interactions and negatively correlated with parenting stress.

#### Retention

Participant retention rates throughout data collection (i.e., pre-testing, post-testing, and follow-up) and throughout SFP (10-14) sessions were directly correlated with the degree of adolescent substance abuse, education level, material quality, recruitment quality (*Spoth, Clair, et al., 2007), and the restrictiveness of attitudes toward adolescent alcohol use (*Skärstrand et al., 2009). Retention rates were inversely correlated with socioeconomic level (*Spoth, Goldberg, & Redmond, 1999), as well as the level of parental responsiveness and affection toward their children (*Skärstrand et al., 2009). One article cited retention rates varying from 69 to 72% (*Mason et al., 2017). Another article indicated strategies used to guarantee retention: serving refreshment immediately before the session begins; availability of transportation and childcare; and holding all the sessions in schools, community centers, churches, and/or clinics close to the families’ residences (*Riesch et al., 2012).

The dropout rate, in turn, was calculated by comparing the number of families who participated in the pre-test and the last data collection. By estimating the numbers given by the articles, the minimum number of families in the last data collection was 12, and the maximum was 562 (M = 173.5; SD = 112.5). This represents an average dropout rate of 34%, for families. In the data from articles that counted parent and child dropout separately, there was a minimum of 13 parents and 15 children, and a maximum of 136 parents and 132 children (M = 61, SD = 48). This represents an average dropout rate of 8% for parents and 7% for children.

##### Implementation barriers and facilitators

The variables that facilitated SFP’s implementation were the presence of childcare, and bilingual, bicultural, or experienced implementation agents (*Orpinas, Reidy, et al., 2014). The variables cited as implementation barriers were the implementation agents’ difficulty to meet prior to the sessions in order to plan them, because they worked in different organizations or had other work demands (*Segrott et al., 2017); family difficulties in showing up for 7 weeks; the session date and time; the perception that the program would require too much family time; very long meetings (2h); beliefs that the family was already doing a good job; the perception that adolescents were not taking risks (*Spoth, Redmond, Hockaday, & Shin, 1996); and the lack of school engagement (*Orpinas, Reidy, et al., 2014).

It is noteworthy that a study applied the qualitative comparative analysis (QCA) to verify the relationship between certain implementation characteristics and SFP effectiveness. The results indicated that a sufficient proportion of trained practitioners (at least 75%), a program size not greater than 12 families, and highly engaged participants are necessary conditions. Additionally, having practitioners who submitted high-quality attendance data, in programs serving at least eight families, is a sufficient condition. When these conditions are present, regardless of other implementation aspects, the program can achieve the targeted outcomes (*Hill et al., 2019).

### Maintenance of SPF (10-14) in Services

Since maintenance at the individual-level results were reported in the effectiveness subsection above, this section would describe maintenance in services—institutions and teams—that implemented SFP (10-14). However, no information was provided by any of the articles.

## Discussion

This study investigated the reach, effectiveness, adoption, implementation, and maintenance (RE-AIM framework) of the 7-session SFP (10-14). The data revealed that studies covering maintenance at setting level and adoption were rare, while the effectiveness, and its maintenance, and implementation dimensions were the most commonly evaluated. Therefore, the evidence of SFP effectiveness, accompanied by evidence for successful and unsuccessful implementation routes, had been significantly accumulated. However, in-depth evaluations that understand the impacts of SFP implementation on organizations and systems are still scarce, which undermines its potential for reach, adoption, and sustainability.

Although the number of participants was widely reported, this information does not allow extracting definitive conclusions regarding the representativeness of the target population in SFP (10-14) implementations worldwide. This gap hinders clear answers regarding the viability of SFP (10-14) in reaching the intended families and if families in most need (e.g., low health literacy) can be reached. This is certainly a clear-cut call for future research, or, at least, for more completeness and detailing on reporting such aspects. Nevertheless, the small sample sizes and the numerous barriers to recruiting them suggest that the reach of SFP (10-14) has been limited and raises doubts about the practicality of its use as part of a system of public policies for families. Some of the proposed solutions to extend the reach and to ease the recruitment of families, such as offering transportation, reimbursing travel costs, paying for participation and meals, would not be feasible in areas with limited resources. This, of course, weakens the program’s potential to be part of the spectrum of integrated public policy services for families in low- and middle-income countries (Mejia et al., 2018). Thus, the data revealed the relative scarcity of studies involving non-Caucasian minority families. Hence, the unanswered questions regarding SFP (10-14)’s viability for minority groups remain.

A large body of evidence about the effectiveness of SFP (10-14) reveals conflicting findings among initial U.S. studies, as well as in more recent European and Latin American studies. On the one hand, the analysis showed SFP (10-14) to be efficacious in the U.S., its country of origin, for the abuse of various substances, in the short- and long-term, and among diverse populations. On the other hand, in the review by Gorman (2017), conflictive results concerning the outcome of substance abuse were found in recent studies in Europe and Latin America. Specifically, while in Germany (*Baldus et al., 2016, *Bröning et al., 2017), Poland (*Foxcroft et al., 2017), Sweden (*Skärstrand et al., 2014), and the UK (*Coombes et al., 2009) substance abuse was unaffected in the short- to medium-term, in Spain, on the other hand, substance abuse was affected (Errasti Pérez et al., 2009), although the Spanish study used a sample of only 26 families. In Latin America, the two studies that investigated the outcome of substance abuse found no significant results (*Corea et al., 2012; Vasquez et al., 2010).

Analysis of non-substance abuse outcomes targeted by SFP (10-14) revealed its effectiveness in the U.S. for parenting practices, depressive symptoms, academic engagement and success, problem resolution, family cohesion, and family relationships. Similarly, Latin American evidence showed improvements in parenting practice (consistently achieved in all studies), parental self-esteem (Vasquez et al., 2010), youth behavior, and couple and family relationships (Mejia et al., 2015). However, European effectiveness evidence was identified only in studies with specific methodological characteristics: a comparative analysis of high and low risk families (*Bröning et al., 2017), small sample sizes (Coombes, Allen, & McCall, 2012; Errasti Pérez et al., 2009), and samples comprising young people with behavior problems (*Coombes et al., 2009).

Thus, three questions can be raised about the difference in effectiveness between the U.S., on the one hand, and Europe and Latin American, on the other hand. One of them pertains to SFP’s adaptation, since a program would supposedly not “work” the same way everywhere. The second relates to the sample size, consistently lower in the European and Latin American studies versus the American ones. Hypothetically, the divergence could be due to the greater margins of error for the smaller sample sizes. The third addresses the time elapsed between the implementation and the last follow-up (and last data collection). The follow-up time varied from 6 to 36 months in non-American studies, while those from the U.S. accompanied participants from the 6^th^ grade (11–12 years old) to adulthood (21 years). This begs the question: how much time is needed for the manifestation of results? For which the answer may be “many years.”

No iatrogenic effects were identified, suggesting that, despite the controversial outcomes achieved, SFP (10-14) is not harmful. This is unsurprising, given the robust theoretical bases of SFP (10-14) and its change mechanism (Kumpfer, 2014). Therefore, implementations delivered with fidelity to its theory of change seem to be free of harmful effects. Nonetheless, public health interventions may have unintentional adverse effects. These are rarely observed, described, or discussed (Lorenc & Oliver, 2014). The study of these effects allows not only the detection of harm, but also the mechanisms related to it, which allows avoiding them in future interventions (Bonell, Jamal, Melendez-Torres, & Cummins, 2015).

The few studies that have analyzed both adoption and maintenance of SFP (10-14) in services revealed that these dimensions are influenced by three factors: (1) financial resources to sustain the program, (2) available conditions for incorporating SFP (10-14) into the service routines, and (3) the recruitment and maintenance not only of agents possessing experience with the target public and good language skills, but also of their motivation. These factors matched the findings from the literature which indicate that both adoption and maintenance are affected by the organization’s preparation (Spoth et al., 2015), planning, and fundraising operations (Cooper, Bumbarger, & Moore, 2013); the leadership and openness of the implementation agents to change, such as modifying their work routine upon adopting SFP (10-14) (Chilenski, Olson, Schulte, Perkins, & Spoth, 2015; Rogers, 2002); and compatibility of the intervention with the values and initiatives of the agents (Rogers, 2002). These are all recommended for maximizing the adoption and maintenance of health interventions (Gaglio et al., 2013).

However, clear conclusions around the adoption rate of SFP (10-14) by services and implementation agents, as well as maintenance within those services, could not be extracted from the findings of this review. Two questions can be addressed to understand this scarcity. First, most of the articles were controlled trials, thus, focused on effectiveness and implementation domains. Therefore, dimensions related to institutional aspects, such as adoption and maintenance, were, unsurprisingly, less reported. Second, the shortage of adoption and maintenance at the setting-level studies is in accordance with the findings of other reviews that used the RE-AIM framework, either on family interventions (Isaacs, Roman, Savahl, & Sui, 2018) or on other health issues (Cuthbert et al., 2017; Gaglio et al., 2013; Jankowski et al., 2014; White, McAuley, Estabrooks, & Courneya, 2009). Moreover, the scarcity of evidence about adoption and maintenance seems to be a challenge not only for SFP (10-14), but also for the field of health interventions in general. Systematic reviews have identified this same gap due to underdeveloped attention to such aspects in other health interventions (Boersma et al., 2015; Eakin, Bull, Glasgow, & Mason, 2002; O’Brien & Finch, 2014; Schlechter et al., 2016).

SFP (10-14) puts various demands on infrastructure: two rooms; on personnel: caregivers and a minimum of three implementation agents; on equipment: DVD player and TV; and on logistics: refreshments, transportation, incentives, and weekly planning (Kumpfer et al., 1996). Thus, the little data available suggests that adoption and maintenance depend on favorable organizational infrastructure (funding, physical space, and routine incorporation) and human capacities (motivation of implementation agent and culture competency) to meet the logistical demands of the intervention’s implementation. These demands, in turn, can complicate adoption by resource-strapped services, as well as incorporation into their work routine. Together, these factors could impede institutionalization and large-scale implementation, particularly in low- and middle-income countries (Mejía et al., 2019).

Analysis of the implementation dimension showed that SFP (10-14) has been implemented with high fidelity, which relates to the characteristics and number of participants in groups, as well as the implementation agents’ characteristics and skills; with a positive relationship between cost and effectiveness in U.S. implementations; with adaptations generally restricted to the superficial structure (except the Swedish version); and the varying levels of engagement and retention associated with three aspects: meeting attendance logistics, belief in intervention quality, and the families’ perceptions of the risk of alcohol and drug abuse by their children. These aspects coincide with those identified as implementation barriers which, in addition to familial barriers, include organizational ones, such as deficits in planning, engagement, and time availability of the implementation team and implementation environment. These findings are consistent with others that reveal factors such as resource scarcity (restricted time, high workload) and weak collaboration as frequent and relevant implementation barriers (Fischer, Lange, Klose, Greiner, & Kraemer, 2016; Winstanley, Clark, Feinberg, & Wilder, 2016).

The intervention cost was evaluated in different ways, though only in American studies, indicating a positive cost-effectiveness relationship, which should encourage adoption and maintenance of the program as a public policy. However, implementation cost data were absent in other countries. This indicator always comes up when addressing fundraising and the public agenda for the adoption and maintenance of the program at a government level (Claxton et al., 2015; Neumann, Sanders, Russell, Siegel, & Ganiats, 2016). In the same way, the tracking of the intervention planning and preparation time, absent from all the studies, is fundamentally important, as it comprises the personnel cost calculation of SFP (10-14) and could help answer questions related to its viability, effectiveness, and sustainability.

## Conclusion

In conclusion, the findings have shown evidence of the effectiveness and maintenance of effects at the individual-level in the U.S.; evidence of the effectiveness in familial outcomes in Latin America, where more assessments about primary outcomes and maintenance are needed; and controversial evidence of the effectiveness in Europe. Additionally, the small number of studies and indicators analyzed regarding adoption, reach, and maintenance at the setting level indicate scarce evidence of feasibility and sustainability of SFP (10-14) worldwide.

The existing data, particularly regarding reach, implies there are barriers to the viability of the intervention, questioning if its use of large-scale implementation initiatives is practical, particularly where resources are scarce. Analyzing this situation, from the viewpoint of the stages of knowledge translation, reveals that notwithstanding the substantial global investment and incredible progress in research on the intervention, other SFP (10-14) studies can only be considered early Type 2 Translation, in other words, understanding the processes and mechanisms that lead to the adoption, large scale implementation and sustained in new contexts, of an intervention that is effective in a given context (*Spoth, Rohrbach, et al., 2013; Spoth, Trudeau, et al., 2013). The small sample sizes and low number of studies that have analyzed SFP (10-14) adoption, reach, and sustainability clearly demonstrate that much remains to be done to understand how the program will perform under a large-scale implementation and transferred to social policies and systems. It is crucial to build capacity in order to favor reach (Mauricio et al., 2018), support systems-oriented scaling up of SFP (10-14) or other evidence-based preventive interventions (*Spoth, Rohrbach, et al., 2013; Spoth, Trudeau, et al., 2013), mainly in scarce resources settings (Mejía et al., 2019). This aligns with the state-of-the-art in prevention science, in general (Fishbein et al., 2016; *Spoth, Rohrbach, et al., 2013; Spoth, Trudeau, et al., 2013). Furthermore, even if investments in the prevention area are mostly provided to Type 1 Translation (*Spoth, Rohrbach, et al., 2013; Spoth, Trudeau, et al., 2013), i.e., understanding the role of putative risk and protective factors in the behavior of young people, the wide range of cultural and social factors may require revision and update every time change of setting or target population is performed.

This study presents some limitations in the interpretations of its results. The exclusion of studies published in books, on internet sites, and in dissertations, theses, reports, as well as in articles in other languages may have resulted in loss of evidence. The heterogeneous manner used to measure the outcomes, such as the instruments utilized, research design, and data analysis strategies—obtaining the missing data, for example—made synthesizing the effectiveness results challenging. Moreover, the methodological rigor utilized by the studies was not examined, which is particularly important to assess the effectiveness of the interventions. For these reasons, the effectiveness findings were treated using a more descriptive manner rather than applying a meta-analytical approach, which could be done in future studies. Lastly, considering the consistently lower sample sizes of European and Latin American effectiveness studies, when contrasted with the U.S. studies, the results and discussion comparing them should be carefully executed.

The need for future studies clearly emerged from the findings of the present review. First, the research agenda should include studies that examine the adoption, reach, and maintenance of SFP (10-14) at an organizational level, objectives which are conspicuously rare at this time. Second, economic evaluation of the implementation in low- and middle-income countries, as well as effectiveness analysis for minority families, could help address questions about SFP (10-14)’s viability, effectiveness, and sustainability. Third, when ongoing randomized controlled trials in Latin American are completed and can offer data about effectiveness that enable meta-analysis, this would be an important study design to be performed. Fourth and last, the discordant findings among the initial studies from the U.S. and more recent studies from Europe and Latin America, express the need to use robust designs in terms of internal and external validity to examine SFP (10-14) effectiveness in new settings. Contextual aspects—geographic, sociocultural, legal, political, epidemiological, socioeconomic, and ethical—should be addressed in an integrated manner, through the implementation process, as they critically influence the effectiveness of a program (Pfadenhauer et al., 2017). Therefore, having longitudinal evaluations based equally on quantitative and qualitative methods that explain which situations and mechanisms predict patterns of program success and failure may be particularly appropriate (Pawson & Tilley, 1997).

## Supplementary Information

**Additional file 1:Supplementary Appendix.** Number of included papers supplying information about the RE-AIM dimensions.

## Data Availability

Not applicable.

## Abbreviations

ATOD: Alcohol, tobacco, and other drugs
CG: Control group
EG: Experimental group
FM: Family Matters
MSFP: Mindfulness-enhanced Strengthening Families Program
PDFY: Preparing for the Drugs Free Years
PROSPER: Promoting School-University Partnerships to Enhance Resilience
QCA: Qualitative comparative analysis
SFP: Strengthening Families Program
UNODC: United Nations Office on Drugs and Crime
