# Supplementary material for: Beyond effectiveness of the Strengthening Families Program (10-14): a scoping RE-AIM-based review
Source: Psicol Reflex Crit. 2021 Jun 15;34:16. doi: 10.1186/s41155-021-00182-z (PMC8206301; doi:10.1186/s41155-021-00182-z)
Supplement: Supplementary file 1 — Additional file 1:Supplementary Appendix. Number of included papers supplying information about the RE-AIM dimensions. [file 41155_2021_182_MOESM1_ESM.docx]

*Supplementary Appendix.* Number of included papers supplying information about the RE-AIM dimensions.

| **Criteria of each RE-AIM dimension** | **N (%)** |
| --- | --- |
| **Reach.** |  |
| Met All criteria; | 1 (2) |
| Exclusion criteria: % of the population excluded or characteristics of the population not targeted by the intervention; | 7 (11) |
| Percentage of individuals who participated in the intervention based on a valid denominator; | 16 (25) |
| Characteristics of participants and/or non-participants; | 51 (78) |
| Use of qualitative and quantitative methods to understand the recruitment/invitation: instruments, barriers, and enablers. | 24 (37) |
| **Effectiveness.** |  |
| Met all criteria; | 0 |
| Measurements of primary and/or secondary outcomes or objectives; | 43 (66) |
| Measurements of unforeseen or collateral outcomes. | 0 |
| **Adoption – setting level.** |  |
| Met all criteria; | 0 |
| Service exclusion criteria and/or % of services excluded; | 0 |
| % of services approached that adopted the intervention; | 0 |
| Characteristics of participating services (GC and GE) compared to (1) non-participating services or (2) relevant resource data; | 1 (2) |
| Use of qualitative and quantitative methods to understand the adoption level of the service: instruments, barriers and enablers. | 1 (2) |
| **Adoption by implementation agents (IA).** |  |
| Met all criteria; | 0 |
| IA exclusion criteria and/or % of IA excluded; | 0 |
| % of IA approached that adopted the intervention; | 0 |
| Characteristics of IA that adopted the intervention compared to (1) non-participants or (2) typical Human Resources (HR); | 2 (3) |
| Use of qualitative and quantitative methods to understand the IA adoption level: instruments, barriers and enablers. | 1 (2) |
| **Implementation.** |  |
| Met all criteria; | 0 |
| Adaptations made to the intervention; | 5 (8) |
| Fidelity; | 23 (35) |
| Uncounted intervention time; | 0 (0) |
| Financial cost or economic analysis of the intervention; | 4 (6) |
| Percentage of dose delivered and received; | 8 (12) |
| Engagement; | 6 (9) |
| Retention; | 5 (8) |
| Context; | 0 (0) |
| Barriers and enablers. | 5 (8) |
| **Maintenance – setting level.** |  |
| Met all criteria; | 0 |
| Whether the program continued running, with additional offerings, after the first follow-up offering; | 0 |
| Use of qualitative and quantitative methods to understand the institutionalization of the program: instruments, barriers and enablers. | 0 |
| **Maintenance – individual level.** |  |
| Met all criteria; | 0 |
| Measurements of primary and/or secondary outcomes or objectives a minimum of 12 months after the end of the intervention; | 26 (40) |
| Measurements of unforeseen or collateral outcomes a minimum of 12 months after the end of the intervention. | 0 |
